# CD44 Expression in Oro-Pharyngeal Carcinoma Tissues and Cell Lines

**DOI:** 10.1371/journal.pone.0028776

**Published:** 2012-01-05

**Authors:** Abirami Rajarajan, Angela Stokes, Balvinder K. Bloor, Rebecca Ceder, Hemini Desai, Roland C. Grafström, Edward W. Odell

**Affiliations:** 1 Molecular Oncology, Department of Oral Pathology, King's College London, London, United Kingdom; 2 Institute of Environmental Medicine, Division of Molecular Toxicology, Karolinska Institutet, Stockholm, Sweden; 3 VTT Technical Research Centre of Finland, Medical Biotechnology, Turku, Finland; University of Birmingham, United Kingdom

## Abstract

Expression of CD44, a transmembrane hyaluronan-binding glycoprotein, is variably considered to have prognostic significance for different cancers, including oral squamous cell carcinoma. Although unclear at present, tissue-specific expression of particular isoforms of CD44 might underlie the different outcomes in currently available studies. We mined public transcriptomics databases for gene expression data on CD44, and analyzed normal, immortalized and tumour-derived human cell lines for splice variants of CD44 at both the transcript and protein levels. Bioinformatics readouts, from a total of more than 15,000 analyses, implied an increased CD44 expression in head and neck cancer, including increased expression levels relative to many normal and tumor tissue types. Also, meta-analysis of over 260 cell lines and over 4,000 tissue specimens of diverse origins indicated lower CD44 expression levels in cell lines compared to tissue. With minor exceptions, reverse transcribed polymerase chain reaction identified expression of the four main isoforms of CD44 in normal oral keratinocytes, transformed lines termed DT and HaCaT, and a series of paired primary and metastasis-derived cell lines from oral or pharyngeal carcinomas termed HN4/HN12, HN22/HN8 and HN30/HN31. Immunocytochemistry, Western blotting and flow cytometric assessments all confirmed the isoform expression pattern at the protein level. Overall, bioinformatic processing of large numbers of global gene expression analyses demonstrated elevated CD44 expression in head and neck cancer relative to other cancer types, and that the application of standard cell culture protocols might decrease CD44 expression. Additionally, the results show that the many variant CD44 exons are not fundamentally deregulated in a diverse range of cultured normal and transformed keratinocyte lines.

## Introduction

CD44 plays an important role in many cell processes including growth, survival, differentiation, and motility [1], by binding ligands such as hyaluronan, osteopontin, collagens, growth factors, and matrix metalloproteinases. *CD44* is a large, highly conserved, and complex gene containing 19 exons located on human chromosome 11 [2] and mouse chromosome 2 [3]. The predominant *CD44* transcript (exons 1–5 and 16–20) produces the standard or haemopoietic form of CD44 (CD44s or CD44h) [4]. The remaining exons (6–15) are variably spliced and referred to as variant exons 1–10 (v1-10). In humans, exon 6 (v1) contains a stop codon and therefore, the first transcribed variant exon of human *CD44* is exon 7 (v2) [5]. The differential utilisation of these variant exons, commonly in tandem blocks rather than independently, generates multiple CD44 variants and these subsequently undergo post-translational modification. The 3′ expression arrays that form the majority of the current publicly available gene expression databases cannot discriminate between alternatively spliced variants of CD44 because the transcripts have identical 3′ ends, however, many studies have been published using CD44v specific primers and antibodies for the detection of CD44 spliced variants.

CD44 is constitutively expressed by almost all cell types [6], [7], but CD44 variant exons (CD44v) have a more restricted distribution and, possibly, function. *CD44* alternative splicing is related to neoplasia and metastasis in many cancers [8]. However, evidence for the role of CD44v in oral squamous cell carcinoma (OSCC) is conflicting. Like keratinocytes of the epidermis, mucosal keratinocytes express all variant exons constitutively [9], [10], [11], [12], consistent with a role for CD44 in structural integrity. Immunohistochemical techniques have suggested that a loss of expression of variant exons in OSCC correlates with higher histological grade and metastasis. Although studies differ, the most frequently implicated variant exons in oral and head and neck squamous cell carcinoma (HNSCC) are v4, 7, 8, and 9 [11], [13], [14], [15].

We have previously shown overexpression of *CD44v* transcripts in OSCC primary tumour samples compared with normal oral mucosa [16]. The splicing patterns of tumour samples and of OSCC cell lines were very similar to normal oral epithelium and normal oral keratinocytes [12], [16]. However, the transcripts were only variably translated to protein [12]. The assembly of public gene expression data into databases, such as the In Silico Transcriptomics database (IST) and the Human Gene Expression Map (HGEM), provide means to assess the expression of genes across thousands of normal and cancer tissue samples. Additionally, *CD44* gene expression levels can be compared among various cancer types, other diseases, and cell lines.

In the present study, we mined transcriptomics databases for *CD44* expression with particular reference to oral and pharyngeal squamous cell carcinoma (OPSCC). Additionally, we assessed *CD44v* transcription and expression in a range of normal, immortalized and tumour-derived epithelial cell lines, including previously uncharacterized lines from three paired primary tumour and lymph node OPSCC metastases.

## Materials and Methods

### Ethics Statement

The isolation and culture of the normal oral keratinocytes used in this study was approved by the ethical committee at the Karolinska Institutet, Reference number 01-092. Each patient provided informed written consent.

### Assessment of *CD44* expression in the In Silico Transcriptomics (IST) database and the Human Gene Expression Map (HGEM) database

*CD44* expression was assessed from bioinformatics-based mining of the IST database (compiled from 9783 Affymetrix gene expression analyses in 43 normal tissues, 68 cancer types and 64 other diseases) and the HGEM database (compiled from 5372 gene expression analyses from 369 different cell and tissue types, disease states and cell lines). Both databases are publically available at http://www.genesapiens.org and http://www.ebi.ac.uk/gxa/experiment/E-MTAB-62, respectively [17], [18].

Applying the IST database, tissue and disease-wide expression profiles were generated for *CD44* with samples represented on the x-axis (normal, cancer, and other diseases) and relative gene expression values on the y-axis. Applying the HGEM database, statistical analyses of six probes on the Affymetrix HG-U133A array that target the *CD44* gene were derived across 96 ‘biological groups’ (cell or tissue types with more than 10 biological replicates among the 369 states) and 4 ‘meta groups’ to assess the expression differences relative to normal, neoplastic, disease and cell line phenotypes.

### Maintenance and culture of primary and secondary cell lines

Cell culture reagents were from Sigma-Aldrich, Gillingham, Dorset, UK unless otherwise indicated. Normal oral keratinocytes (NOK) were obtained from healthy, non-smoking donors undergoing maxillofacial surgery (approved by the ethical committee at the Karolinska Institutet) and cultured as previously described [19]. Briefly, primary cell cultures were derived from tissue digestion with trypsin overnight at 4°C, and the mixture was resuspended in a serum-free epithelial medium with high levels of amino acids (termed EMHA) or Keratinocyte-SFM media (Gibco, Invitrogen Corporation, Paisley, UK) and seeded onto dishes that had been pre-coated with fibronectin/collagen. These media were used interchangeably without detectable differences in growth or outcome of the experiments. Cultures were transferred onto regular tissue culture plastic at approximately 75% confluence, and cells in passage 2 were used in the experiments.

HPV immortalised epidermal keratinocytes (DT) were kindly provided by Prof. I. M Leigh. Three cell line pairs HN4/HN12, HN22/HN8, HN30/HN31, kindly provided by Dr. A Yeudall, were established from primary tumour and lymph node metastases from three individuals with squamous cell carcinomas of the tongue, epiglottis, and pharynx, respectively [20]. DT cells, the three cell line pairs, and the spontaneously immortalised epidermal human skin cell line HaCaT [21] were maintained in Dulbecco's modified Eagle's medium (DMEM) supplemented with 10% foetal calf serum (FCS), 2 mM glutamine, and 100 µg/ml penicillin/streptomycin. Two cell lines, OSC-19 and -20, isolated from squamous cell carcinomas of the tongue that metastasised to a cervical lymph node [22], and were metastatic in nude mice, were maintained in a 3∶1 ratio of DMEM∶F-12 Ham's medium (Sigma) supplemented with 10% FCS, 10 ng/ml epidermal growth factor (EGF), 0.5 µg/ml hydrocortisone, 5 µg/ml transferrin, 5 µg/ml insulin, 1.8×10^−4^ M adenine, 1×10^−10^ M cholera toxin, 100 units/ml penicillin, and 100 µg/ml streptomycin. Namalwa, an Epstein-Barr virus immortalised Burkitt's lymphoma line [23], was grown in RPMI-1640 medium supplemented with 10% FCS and used as a negative control.

### Reverse transcription-polymerase chain reaction (RT-PCR)

RNA was extracted from cells using RNeasy spin columns (Qiagen, Crawley, West Sussex, UK) as previously described [12]. Synthesis of first strand cDNA from 2.5 µg of total RNA was carried out using 12.5 ng of Oligo (dT) primers (Invitrogen Ltd, Paisley, UK), and denatured at 70°C for 10 minutes. The samples were then placed on ice for 5 minutes, before adding 1× first strand buffer, 10 mM DTT (Dithiothreitol; Invitrogen), and 500 µM of each dNTP. After a 2 minute annealing incubation at 42°C, 10 units of Superscript II reverse transcriptase (Invitrogen) were added and further incubated for 50 minutes at 42°C. A final 15 minute incubation at 70°C terminated the reaction, and the resultant cDNA was stored at −20°C.

Hot start PCR was performed on cDNA primers for total *CD44* (*CD44* P1 and P2 primers), individual variant exons (*CD44* P1 and exon-specific reverse primers for v1 to v10) [24], and β actin [25] (see Table 1 for primer sequences). 5 µl of cDNA, 20 mM Tris-HCl (pH 8.4), 50 mM KCl, 1× Q solution (Qiagen), 200 µM of each dNTP, and 20 µM of each 5′ and 3′ primer was denatured for 5 minutes at 95°C, followed by a 10 minute 72°C elongation phase during which 0.25 units of Taq DNA polymerase (Qiagen) were added. Amplification of DNA was performed in a Perkin-Elmer GeneAmp PCR System 2400 using 35 cycles of denaturation for 1 minute at 95°C, annealing for 1 minute at 57°C, and elongation for 2 minutes at 72°C. Reaction products were resolved by electrophoresis on a 1.5% agarose gel. To confirm specificity of amplification, PCR products were purified (QIAquick, Qiagen), sequenced (Perkin Elmer ABI 377), and their identity confirmed using BLAST software from NCBI (Zhang and Madden, 1997).

**Table 1:** RT-PCR Primer Sequences.

Primer	Primer Length (bases)	Sequence (5′ to 3′ direction)
***CD44*** ** P1 F**	21	TCCCAGTATGACACATATTGC
***CD44*** ** P2 R**	20	CACCTTCTTCGACTGTTGAC
***CD44*** ** v1 R**	20	CTGTGAATTACCAAACCAGG
***CD44*** ** v2 R**	20	TGCTGTAGCACTAGTGCTCA
***CD44*** ** v3 R**	20	TCATTTGGCTCCCAGCCTGC
***CD44*** ** v4 R**	21	TTGTCTGAAGTAGCACTTCCG
***CD44*** ** v5 R**	21	GTTCCAGTTTCCTTCATAAGC
***CD44*** ** v6 R**	20	CCCACATGCCATCTGTTGCC
***CD44*** ** v7 R**	21	AATCAGTCCAGGAACTGTCCT
***CD44*** ** v8 R**	21	GTGTTTGGATTTGCAGTAGGC
***CD44*** ** v9 R**	21	GTCTTTATCTTCTTCCAAGCC
***β actin*** ** F**	20	CGTACCACTGGCATCGTGAT
***β actin*** ** R**	21	GTGTTGGCGTACAGGTCTTTG

Key: F = Forward Primer, R = Reverse Primer.

For *CD44v*, the *CD44* P1 F primer was combined with the exon-specific reserve primer.

### Immunocytochemistry (ICC)

Cells in log growth phase were detached by trypsinisation and resuspended in medium with 10% FCS at 2×10^4^ cells/ml. 20 µl aliquots of cell suspension were applied to APES treated polytetrafluoroethylene-coated microscope slides and grown for 2 days at 37°C in 5% CO_2_.

Slides were blocked in 3% hydrogen peroxide in 50% methanol for 30 minutes to stop endogenous peroxide activity and then in 20% normal rabbit serum (DAKO Ltd, Ely, Cambridgeshire, UK) for 10 minutes to block non-specific binding. They were then incubated for 60 minutes with primary antibody (see Table 2), followed by 30 minutes each in secondary biotinylated rabbit anti-mouse antibody (1∶300, DAKO) and streptavidin-biotin horseradish peroxidase (1∶50, DAKO). Binding was visualised with 3,3′ diaminobenzidine (DAB; DAKO Ltd, UK) and slides were counterstained with haematoxylin (VWR International Ltd, Lutterworth, Leicestershire, UK). Isotype-matched irrelevant antibodies were used as negative controls. All of the antibodies were reported to be suitable for ICC by the manufacturers, and the optimum titre was determined separately.

**Table 2:** Primary Antibodies.

				Final Concentration (µg/ml)		
Antibody	Clone/Code	Source	Isotype	ICC	WB	FC
**β actin**		1	IgG	-	2	-
**CD44S**	2C5	3	IgG2A	1	2	16.7
**Exon v3**	3G5	4	IgG2b	2	2	16.7
**Exon v4**	VFF11	4	IgG1	1	2	16.7
**Exon v5**	VFF8	4	IgG1	5	2	16.7
**Exon v6**	2F10	4	IgG1	1	2	16.7
**Exon v7**	VFF9	4	IgG1	5	2	16.7
**Exon v7/8**	VFF17	4	IgG2b	2	0.5	16.7
**Exon v9**	441V	5	IgG1	2	2	16.7
**Exon v10**	VFF14	4	IgG1	20	2	90

1 – Santa Cruz, USA, 2 – Dako Ltd, UK, 3 – R&D Systems, UK, 4 – Serotec, UK, 5 – AmsBiotech (Europe) Ltd.

### Western blotting

Cells were lysed for 10 minutes on ice with NP40 buffer and the postnuclear supernatant was stored at −70°C. Protein concentrations were determined by Coomassie blue dye-binding (Bio-rad Laboratories, Hemel Hempstead, Hertforshire, UK). Then 25 µg of protein was boiled for 5 minutes with gel-loading buffer and resolved in an 8% polyacrylamide-SDS gel and transferred to immobilon-P membrane (Millipore UK Ltd, Croxley Green, Watford, UK) using a semi-dry method. Non-specific binding sites were blocked with 5% dried skimmed milk or BSA in PBS containing 1% Tween 20. Blots were probed with primary antibodies against total CD44, individual variant exons, or β actin (Table 2), and visualised using horseradish peroxidase-conjugated (HRP) secondary antibody (1∶5000–1∶10,000 sheep anti-mouse, GE Healthcare, Amersham, Buckinghamshire, UK) for CD44, or (1∶1000 goat anti-mouse, DAKO Ltd, UK) for β actin and enhanced chemiluminescence (ECL, Amersham). All the primary antibody concentrations were titrated in the range 2–10 µg ml^−1^ using test blots, to determine the optimum concentrations.

### Flow cytometric analysis

Approximately 1×10^66^ cells were washed and resuspended in 100 µl PBS containing 2.5% FCS and incubated for 1 hour at 4°C with the primary antibody (Table 2) at concentrations determined by control titration assays. Cells were then incubated with FITC-conjugated goat Fab′ fragments directed against mouse IgG (1∶5,000, Cappel Laboratories, Malvern, PA, USA) for 30 minutes at 4°C. After washing, the cells were fixed using 2% paraformaldehyde in PBS, and 10,000 events were analysed by flow cytometry on a FACScan (Becton Dickinson UK Ltd, Littlemore, Oxford, UK). Background/non-specific staining was subtracted using controls stained with irrelevant isotype-matched antibody at the same concentration.

## Results

### *CD44* expression in normal and disease tissues

**Figure 1:**
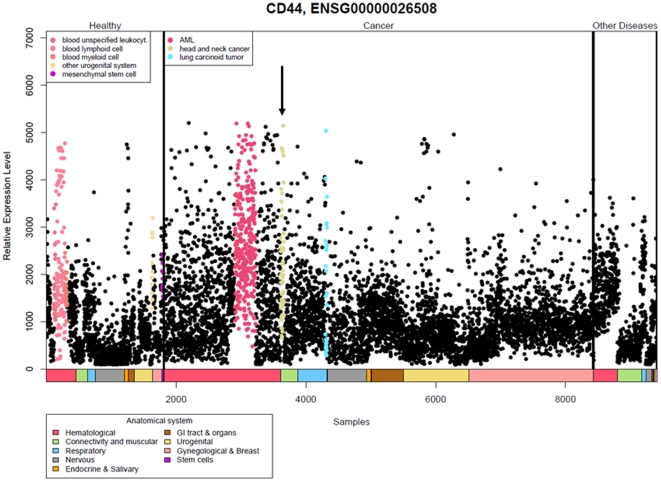
Expression profiles of CD44 from the In Silico Transcriptomics database. Each sample is represented by a dot and colour coded as in the ‘anatomical systems box’. The samples are divided into three sections; healthy, cancer and other diseases. Samples with higher average expression levels are indicated in colour in the figure, and the corresponding tissues are displayed in the top left of the respective sections, i.e., higher than average expression for CD44 was observed in tumour samples from acute myeloid leukaemia, head and neck cancer (indicated by arrow) and lung carcinoid tumour.

The expression of *CD44* was assessed initially at transcript level by mining the IST database that includes tissue samples from normal non-neoplastic tissues, malignant neoplasms and other diseases (Figure 1). Relative plotting displayed elevated expression levels of *CD44* in head and neck cancer as well as in acute myeloid leukemia (AML) and lung carcinoid tumors relative to 65 other cancer types (see box in the “Cancer” compartment). The relative expression level among specimens from these cancer types differed within a 10-fold range. Above average expression was also noted for five types of normal, non-neoplastic tissue, although notably, not for normal reference samples to HNSCC (see box in ‘Healthy’ compartment). The ‘Other diseases’ compartment did not indicate elevated *CD44* expression (Figure 1).

Next, we assessed *CD44* expression in the HGEM (Table 3). Six probes targeting *CD44* displayed significantly increased expression in the oral squamous cell carcinoma group relative to 95 other biological groups. Due to the inclusion of 369 cell line samples in the HGEM, we next compared this entity versus normal, neoplastic and diseased tissue specimens. The normal and neoplasm groups showed increased expression of *CD44* for between five and six probes, respectively, whereas decreased expression of *CD44* was found for five out of six probes in the disease and cell line groups (Table 3). The cell line group included only one line from the head and neck region, i.e., the tongue squamous cell carcinoma line Cal27, which showed elevated expression for all probes relative the other cell lines (data not shown).

**Table 3:** Meta-analyses of CD44 Expression in the Human Gene Expression Map^a^.

CD44 (probe id)	Oral squamous cell carcinoma^b^	Normal^c^	Neoplasm^c^	Disease^c^	Cell line^c^
204489_s_at	**Up**	**Up**	**Up**	**Down**	**Down**
	(1.03*10^−9^)	(2.86*10^−4^)	(3.57*10^−8^)	(0.018)	(1.48*10^−8^)
204490_s_at	**Up**	**Up**	**Up**	**Down**	**Down**
	(<1*10^−10^)	(0.001)	(3.26*10^−6^)	(0.027)	(3.1*10^−6^)
209835_x_at	**Up**	**Up**	**Up**	**Down**	**Down**
	(<1*10^−10^)	(0.004)	(9.57*10^−7^)	(0.033)	(3.38*10^−6^)
210916_s_at	**Up**	**Non d-e**	**Up**	**Down**	**Non d-e**
	(0.002)	(0.11)	(0.003)	(1.15×10^−5^)	(0.369)
212014_x_at	**Up**	**Up**	**Up**	**Down**	**Down**
	(<1*10^−10^)	(0.03)	(3.67*10^−7^)	(0.01)	(4.43*10^−4^)
212063_at	**Up**	**Up**	**Up**	**Up**	**Down**
	(<1*10^−10^)	(<1*10^−10^)	(<1*10^−10^)	(1.55×10^−4^)	(<1*10^−10^)

a CD44 expression assessment in the human gene expression map. The database contains 5372 samples hybridized to the Affymetrix HG-U133A array platform for comparison of the expression levels relative to various biological variables e.g., 96-groups or 4-meta groups. Direction of change (Up, Down or Not differently expressed (non d-e)) and p-values (bracketed) is indicated for the respective “groups” for probes targeting CD44. A p<0.05 was considered significant. For details, see Materials and Methods.

b Assessment of the biological variable “96-groups” that includes the group “oral squamous cell carcinoma” relative to CD44 expression. Each of the 96-groups contains at least 10 biological replicate samples. The OSCC group contains 23 samples from one study [65].

c Assessment of the biological variable “4-meta groups”. The normal group contains 1033 samples, neoplasm 2315 samples, disease 765 samples and cell line 1259 samples.

### Transcription of *CD44* isoforms

**Figure 2:**
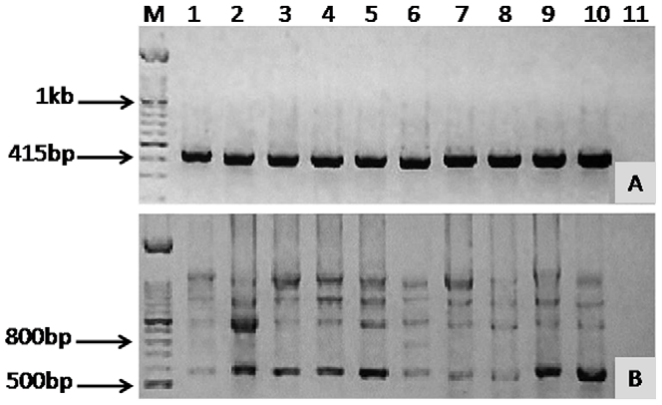
Amplification of *β actin* and *CD44*. RT-PCR was performed, and resolved on a 1.5% agarose gel with a 100 bp ladder. Lanes 1–10 contain NOK, DT, HN4, HN12, HN8, HN22, HN30, HN31, OSC-19, OSC-20. (A), bands at 411 bp are β actin product band. (B), With P1 and P2 primers, bands at 549 bp indicate presence of *CD44s* RNA in all samples. In addition, NOK and all the other lines produce isoform bands consistent with splicing of variant exons (bands at 1 kb, 1.3 kb, and 1.7 kb). M = 100 bp marker, and Lane 11 water control.

Amplification of housekeeping gene *β actin* generated a 411 bp band from all cell lines confirming the integrity of cDNA (Figure 2A). No bands were seen in the negative control.

Using the standard total *CD44* PCR primers, P1 and P2, it can be seen that all cell lines produced a transcript at 549 bp, indicating transcription of *CD44s* (Figure 2B). Additional bands, using these primers, were also seen at approximately 1 kb, 1.3 kb, 1.6 kb, and 1.7 kb, consistent with transcription of *CD44* containing variant exons v8-10, v6-10, v3-10, and v2-10, respectively. A band at 800 bp seen in DT and HN22 cell lines (lanes 2 and 6, respectively) was not compatible with any recognised combination of variant exons. Few, if any, differences were detected between each of the two pairs of cell lines; HN4∶HN12, HN22∶HN8, and HN30∶HN31 (lanes 3∶4, 6∶5, and 7∶8, respectively) apart from the additional band at 800 bp seen in HN22 (lane 6) but not in its metastasis-derived partner HN8 (lane 5).

### Transcription of individual variant exons

**Figure 3:**
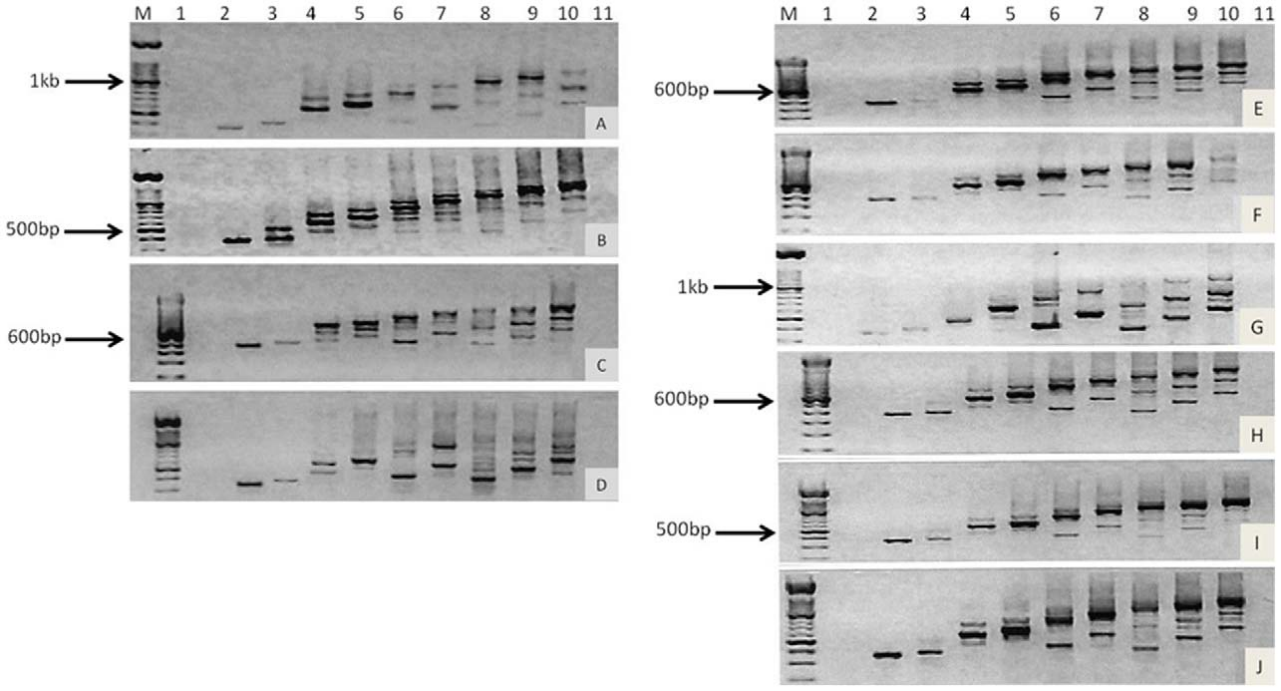
Amplification of *CD44* variant exons. RT-PCR was performed using exon specific primers (lanes 1–10 were exons v1 to v10, respectively), and resolved on a 1.5% agarose gel with a 100 bp ladder. NOK cells (A), DT cells (B), OSC-19 (C), and OSC-20 (D) and three matched primary and metastasis derived OPSCC cell line pairs; HN4 (E) and HN12 (F), HN22 (G) and HN8 (H), and HN30 (I) and HN31 (J) were assessed.

Exon specific PCR amplifies *CD44* from the 5′ end of *CD44s* (constituitive exons 3 to 5) and all of the variant exons spliced to it. For example, the variant exon v4 reverse primer, together with the standard forward primer P1, will amplify exons 3–5 and v2-4 (v1 not transcribed in humans). When the products from each variant exon primer were resolved in sequence on a gel, a stepwise sequence of bands of progressively larger size was observed (Figures 3A and B) reflecting sequential exon transcription. Additional linear diagonal patterns of bands of smaller size were also seen, consistent with shorter transcripts containing fewer variant exons.

Overall, there was a tendency to start variant transcription at exon v2, v3, v4, v6, or v8. Variant exon 1 was not transcribed at all consistent with other known sequences (Table 4, Figure 3A–J). It can be seen that all cell lines produced four similar transcripts; v2-10, v3-10, v6-10, and v8-10, except HN22, which lacked v3-10. The next most common transcript was v4-7 in HN8, HN12, HN30, HN31, and OSC-19 cells, then v7-10 in DT, HN22, HN31, and OSC-20 cells, followed by v4-6 in NOK, HN22, and OSC-20 cells. Some transcripts were identified in only a minority of cells, such as a long transcript v4-10 in DT cells, and a short minor transcript v9-10 in OSC-20 cells.

**Table 4:** Summary of CD44 variant transcripts.

Cells	*CD44* Transcripts								
	v2-10	v3-10	v4-10	v6-10	v7-10	v8-10	v9-10	v4-7	v4-6
**NOK**	•	•		○		○			○
**DT**	•	•	○	○	○	○			
**HN4**	•	•		○		○			
**HN12**	○	•		○		○		○	
**HN22**	•			•	○	•			○
**HN8**	•	•		•		•		○	
**HN30**	•	•		○		○		○	
**HN31**	•	•		•	○	•		○	
**OSC-19**	•	•		•		•		○	
**OSC-20**	○	•		•	○	•	○		○
• Major band			○ Minor band						

Within each primary-derived and metastasis-derived pair there were no large differences in splicing pattern. Apart from HN22, all produced the four transcripts described above and only one or two short transcripts. A few small differences in transcription patterns were detected within each matched pair. In HN4 the v2-10 transcript was a major band but in HN12 it was minor; HN12 had an additional transcript v4-7; minor transcripts v7-10 and v4-6 were not observed in HN8 and the major transcript v3-10 and minor transcript v4-7 were not found in HN22. One minor transcript found in HN31 (v7-10) was not present in HN30 and v6-10 and v8-10 were minor transcripts in HN30 but major transcripts in HN31.

### CD44 expression assessed by immunocytochemistry (ICC)

**Figure 4:**
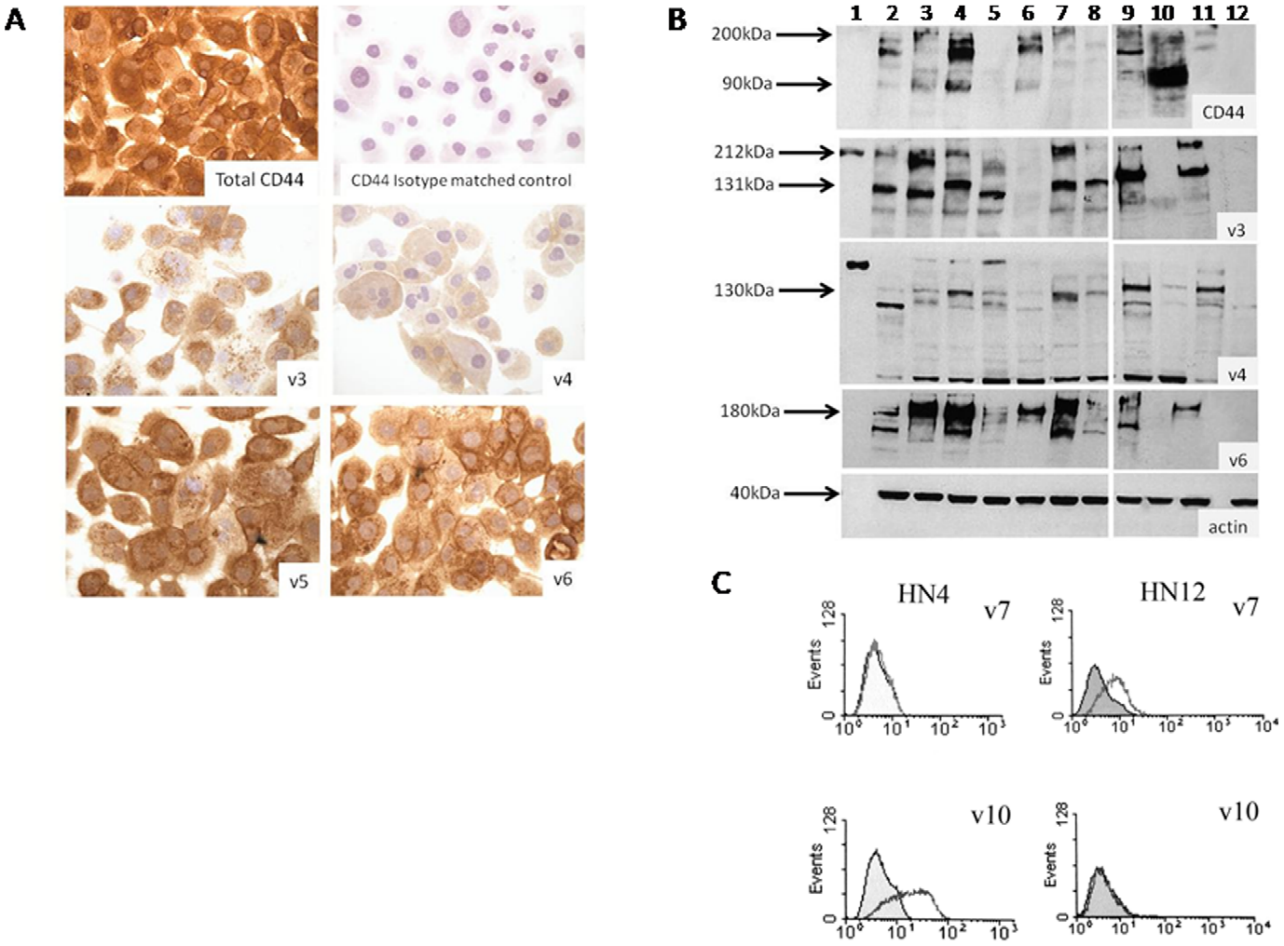
Detection of total CD44 and variant CD44 protein. Representative figures of immunostaining with all cells stained positive in this field (A), western blotting (B), and flow cytometry (C). (A) Immunostaining of HN12 cells for total CD44, an isotype matched control, v3, v4, v5, and v6. (B) Western blotting lanes 1–12 contain MW marker, HaCaT, HN4, HN12, HN8, HN22, HN30, HN31, OSC-19, OSC-20, DT, and Namalwa cells, repectively. (C) flow cytometry results for HN4 and HN12 cells detecting v7 and v10.

In all experiments, isotype-matched negative controls and no-antibody controls showed complete absence of staining. The positive control NOK cells stained for total CD44 and all the variant exons. The staining intensity was scored quantitatively from 0 (absent) to 3. The proportion of cells showing membrane staining was assessed as homogenous (90–100% of cells stained), heterogenous (50–90% of cells stained), occasional (10–40% of cells stained), or sparse (<10% of cells stained). The results from ICC of CD44s and variant exons are summarised in Table 5 and representative images are shown in Figure 4A.

**Table 5:** Summary of total CD44 and variant exons detected by immunostaining, western blotting, and flow cytometry.

Cells	Total CD44	CD44 Variant Exons							
		v3	v4	v5	v6	v7	V7/8	v9	v10
**Immunostaining**									
**NOK**	2–3	3	1–2^S^	3	2–3^H^	2^S^	3	2^H^	3^H^
**DT**	1–3^H^	2–3	0	2–3	1–2^H^	0	2	1–2	1–2^OC^
**HN4**	2–3	2–3^H^	1–2^OC^	1–2^H^	2–3^H^	0	2–3^H^	2–3^H^	2–3^OC^
**HN12**	2–3^S^	2–3^OC^	2–3^OC/S^	2–3^H^	2–3^H^	2–3^S^	2–3^H^	2–3^S^	2^OC^
**HN22**	-	-	-	-	-	-	-	-	-
**HN8**	2–3^H^	3	0	1–2^H^	2–3	0	0	2–3	2^OC^
**HN30**	2–3^H^	2–3	2^H^	2–3	2–3	0	1–3^H^	2–3	2
**HN31**	2–3	1–3	1–2^S^	2–3^H^	2	0	2^OC^	2–3^H^	2^H^
**OSC-19**	2–3	1–3	1–2^H^	3	3	2–3	2^H^	2–3	3^H^
**OSC-20**	2–3	1–2^OC^	0	2^H^	2–3^OC^	0	0	1–2^H^	0
**Western Blotting**									
**DT**	A C D	C D	C	C D	C D	B C	D E	B C	D E
**HN4**	A C D	B D	C D	C D	D	B	D E	B C	D E
**HN12**	A C D	C D	C D	C D	C D	B C	D E	B C	D E
**HN22**	A C D	0	C	C D	D	B C	D E	B C	D E
**HN8**	0	B C	C D	C D	D	B C	D E	B C	D E
**HN30**	C D	C D	C	C D	C D	C	D E	B C D	D E
**HN31**	C D	C D	C	C D	C D	B C	D E	B C D	D E
**OSC-19**	A C D	C D	C	C D	C D	B C	D E	B C D	D E
**OSC-20**	A	0	C	C D	0	B	D E	B C D	D E
**Flow Cytometry**									
**DT**	+ +	+ +	−	+ +	+ +	−	+ +	+ +	+
**HN4**	+ +	+ +	+ +	+ +	+ +	−	+ +	+ +	+ +
**HN12**	+ +	+ +	+ +	+ +	+ +	+ +	+ +	+ +	−
**HN22**	nt	nt	nt	nt	nt	nt	nt	nt	nt
**HN8**	+ +	+ +	+ +	+ +	+ +	+	+ +	+ +	+ +
**HN30**	+ +	+ +	+	+ +	+ +	−	+	+ +	−
**HN31**	+ +	+ +	+ +	+ +	+ +	−	+	+ +	−
**OSC-19**	+ +	+ +	+ +	+ +	+ +	+ +	+ +	+ +	+
**OSC-20**	+ +	+	−	+	+ +	−	+ +	+ +	−

**Immunostaining.** 0 = no staining; 1 to 3 = increasing intensity of membrane staining. ^S^ = sparse (<10% of cells stained); ^OC^ = occasional (10 to 40% of cells stained); ^H^ = heterogeneous (50 to 90% of cells stained); and finally, homogenous staining (90–100% of cells stained) has no superscripted symbol.

NOTE: HN22 cell line failed to grow for analysis.

**Western Blotting.** Band sizes: A = 90 kDa, B = 120–125 kDa, C = 130–170 kDa, D = 180–200 kDa, E = 230–250 kDa, 0 = no expression.

**Flow cytometry.** ++ = 50% increase in MCF, + = >15 <50% increase in MCF, − = no increase in MCF, nt = not tested.

All cell lines stained intensely for total CD44s protein with >50% of cells stained, except for HN12 where only occasional (<10%) cells were stained. All cell lines also stained (>50% of cells stained) for v3, v5, v6, and v9. The next most common transcript was v10, for which only OSC-20 did not stain, and other cell lines had occasional cells staining. Detailed analysis is shown in Table 5.

There were minimal differences, limited to percentages of cells stained, between the protein expression patterns of the paired OPSCC cell lines based on immunocytochemistry. The only clear difference was that for v7, for which HN4 did not stain, while its metastasis-derived partner HN12 stained sparsely.

### CD44 expression by western blotting

Positive control DT and HaCaT cell lines showed positive bands for all blots probed with CD44 antibodies. Negative control Namalwa cell line generated only bands below 60 kDa and 100 kDa respectively, for v4 and v9 identified as false positives on basis of size. Blots probed with antibody to β actin produced the expected 42 kDa band. The results for the cell lines probed with total CD44 and variant CD44 antibodies are summarised in Table 5 and representative blots are shown in Figure 4B.

All cell lines with the exception of HN8 expressed total CD44 and the majority showed multiple isoforms. The greatest number of bands was expressed by the HN12 cells, which showed bands at approximately 145 kDa, 180 kDa, and faint bands at 90 kDa, 130 kDa, and 200 kDa. CD44 v3 was expressed in bands at 90 kDa, 120 kDa, 130 kDa, and a smear at 185–200 kDa, except in OSC-20 and HN22 cell which did not express v3 at all. Significantly there was some differential expression between cells of the 120 and 130 kDa bands; HN4, and HN8 both expressed the 120 kDa band, whereas HN12, HN30, and HN31 all expressed the 130 kDa band.

Variant 4 was expressed mainly as a strong band at 130 kDa but some cells also expressed further bands at 160 and 180–90 kDa (HN4, HN12, and HN8). OSC-20 did not express v6, but the other cell lines all had a smear at 180–190 kDa. Some cell lines also had an additional band at 130 kDa. All cell lines expressed v7 with bands at 120 or 130 kDa, some minor bands were also present at 160 and 100 kDa. Two main bands were also found for v9 at 120–130 kDa, and some cells also expressed another band at 180–190 kDa. No correlation was found for any of these expression patterns between the cell lines of each pair. All remaining variants (v5, v7/8, v10) were expressed by all cell lines but there were no differences in their expression between the cell lines.

### CD44 expression by flow cytometry

The results for flow cytometry are summarised in Table 5 and representative plots are shown in Figure 4C. All cell lines expressed total CD44 with a greater than 50% increase in mean channel fluorescence (MCF). The most frequently undetected variant exon was v7, followed by v10 and v4. As with other techniques OSC-20 was found to express the fewest variant exons. The main differences between the paired samples were that HN4 did not express v7 while its metastasis-derived partner, HN12, lacked v10. Expression of v4 was infrequent in HN30 (>15 <50% increased MCF) but in HN31 it was highly expressed (>50% increased MCF). HN22 could not be analysed by flow cytometry.

## Discussion

Genome-wide transcriptomic profiles have revealed that head and neck cancer is a heterogeneous disease, impeding accurate prognostication and identification of genes contributing to carcinogenesis. Our previous work indicated higher CD44 expression in oral squamous cell carcinoma tissue compared to normal tissue counterparts [16]. To understand whether this increased expression is related to all cancers or just oral and head and neck cancers specifically, we mined the two currently existing major public transcriptomics databases for *CD44* expression.

The assessment of overall more than 15000 gene expression analyses showed that head and neck cancers had elevated expression compared to the majority of other solid cancers, and also, that *CD44* expression levels might decrease from application of culturing protocols (Figure 1 and Table 3). Large variations were noted among all samples and categories in both databases, possibly related to tumour heterogeneity, variable differentiation states, clonal differences or presence of putative stem cell populations. Although our previous limited sample data analysis suggested similar CD44 expression levels in tissues and cell lines for the head and neck region [16], the absence of stromal influences or general culture-dependent enrichments for proliferative, non-differentiated, or potentially even CD44 variant-specific, cell populations might underlie the generally noted lower expression level of CD44 from bioinformatics-based sorting of large numbers of cell lines.

Current bioinformatics-based expression data cannot distinguish isoforms of CD44. On this basis, we explored also the possibility of alternative splicing of CD44 in several cell lines for the head and neck region, including normal, immortalized and tumor-derived lines, the latter including previously uncharacterized derivatives from matched pairs of normal and metastatic tissue. We hypothesised that given the role of *CD44v* in other cancers, that the individual isoforms could also play an integral role in oral cancers.

All the experimental work was performed on cell lines, and so it cannot be interpreted in relation to any possible CD44 positive cancer stem cell phenotype. Expression of CD44 marks a putative squamous cell carcinoma stem cell population, but only a minority of expressing cells with a basal phenotype have stem cell properties [26]. It appears that hyaluronan binding triggers pleuripotential stem cell regulating genes [27] so that CD44 variants would differ in this activity. The role of CD44 and splice variants as stem cell markers in squamous carcinomas remains to be fully confirmed and there are several alternative hypotheses to link such a constitutively and abundantly expressed molecule with stem cell-like properties [28].

### Transcription of total CD44 and individual variant exons

Few studies have used exon-specific primers to study all variant exon-containing *CD44* isoforms in HNSCC, most have used P1/P2 or equivalent primers [9], [29], to detect total *CD44* or *CD44s*. Two studies have used exon-specific primers for *CD44* v3, finding that v3 transcripts are higher in tumour tissues than normal [29] and suggested that it is linked with growth and migration [30] in HNSCC.

All of the OSPCC cell lines in this study transcribed *CD44s* and the four typical keratinocyte isoforms to varying degrees, except HN22, which failed to transcribe v3-v10. In addition, a variety of other variant exon-containing transcripts were identified. However, no major differences were identified between NOK and DT cells, and between each of the paired primary and metastasis derived lines (HN4∶HN12, HN22∶HN8, and HN30∶HN31), and the lines that were metastatic in nude mice (OSC-19, and OSC-20). These findings indicate that the *CD44* variant splicing patterns of these OPSCC lines are very similar to normal keratinocytes, and appear not to be related to their stable metastatic phenotype.

Transcription patterns in OPSCC lines are better preserved than those reported in breast, bladder, colon, and lung carcinomas. These carcinomas have been shown to transcribe a wider range of splice variants [31], more transcripts that exceed 1.5 kb [32], more transcripts with variant exons [33], and to express preferentially one isoform over another [34]. It is intriguing that splicing patterns in OPSCC appear essentially normal despite the fact that the genome of OPSCC shows much more damage than malignant neoplasms at sites not exposed to tobacco and alcohol. It is possible that normal *CD44* expression has a survival advantage to malignant cells and mechanisms might include improved cohesion and variant exon 6 and 7 ligation to osteopontin, which signals for cell survival via the Akt pathway [35].

RT-PCR can distinguish alterations in *CD44* transcription with much greater sensitivity than ICC [31]. However, RT-PCR must be interpreted in conjunction with other techniques because Southern and western blots have revealed that most of the mRNA transcripts are not translated into protein, implying that over expression at the protein level might involve only a minority of the aberrant RNA transcripts [36].

### CD44 expression by ICC

ICC revealed that all OPSCC cell lines expressed CD44s, indicating expression of constitutive exons consistent with matched PCR data. Only HN12, and OSC-19 gave positive staining for the products of all variant exons. The remaining cell lines failed to stain for at least one variant exon. OSC-20 did not stain for the greatest number of variant exons (v4, v7, v8, and v10). The most commonly absent variant exon product was v7, followed by v4, v8, and v10. No relationship could be detected between failure to stain for a particular variant exon and whether the line was derived from primary or a metastatic site. For most cell lines translation did not correlate with transcription as assessed by exon-specific PCR. This might be due to post translational regulation of expression, defects in translation mechanisms, but more likely to be explained by the failure of some antibodies to detect glycosylated isoforms.

Almost all studies of CD44 expression by HNSCC tissue or cells lines have used ICC. However, the literature is characterised by inconsistency. Loss of total CD44 expression has been associated with metastatic spread, and possibly poor prognosis in HNSCC [37], [38], [39], but others have concluded the opposite, that total CD44 expression is associated with metastasis [40], [41], [42].

Similar contradictory findings have been published for variant exon expression. Loss of expression of v2 [43], v3 [44], [45], v4/5 [13], v6 [46], [47], [48], [49], v7/8 [15] and v9 [50], [51] have been claimed to correlate with either progression from normal to dysplasia, or to malignancy, metastasis, or poor survival. Conversely, down regulation of variant exons v5 and v6 [52], and v8-10 [14] has been claimed to be unrelated to progression, metastasis, or poor survival. There is however, general agreement that expression or over-expression of variant exons v5, v6 [53], [54], [55], [56] and from v4 to v9 [12], [57] does not correlate with tumour progression or predict survival or differ between primary and metastatic lesions.

These inconsistencies may be due to differences in the grading systems used for clinical samples, use of different antibodies, and lack of standard criteria to assess the results. However, there are more fundamental problems affecting the assessment of CD44 expression by ICC. Provided positive and negative controls are appropriate, positive staining indicates expression but failure to stain does not necessarily reflect a lack of expression. The binding of monoclonal antibodies directed to CD44 exon-specific epitopes is compromised by the structural variability of the CD44 molecule. Exon assortment and post-translational modification including glycosylation, can mask CD44 epitopes. In addition glycosaminoglycan side chains may disturb more distant epitopes because they are critical in determining the conformation of a molecule [58]. As a result, ICC is prone to false negative results and must be interpreted with caution.

In this study it was found that cells failed to stain most frequently for variant exon v7 followed by v4, v8, and v10. Few studies have been carried out with these antibodies on HNSCC and similar findings have mostly been interpreted as down regulation or lack of expression. One report concluded failure to stain for v7 and v9 by OSCC was associated with reduced survival [11]. Herold-Mende *et al* also concluded that v7, v8, and v10 were markedly down regulated in primary HNSCC and were not detectable in metastatic carcinoma [14]. Lack of v7/8 staining has also been correlated with poor survival [15].

Staining for v4 has been reported to be lost or reduced in dysplastic mucosa [57] and loss of v4/5 in >50% of OSCC metastases [13], 68% of well differentiated tongue SCC's [45] and in 70% of poorly and moderately differentiated OSCC [9]. Loss of staining with v4/5 antibody could indicate down regulation or loss of either v4, v5, or both. Exon v5 has been generally found to be expressed by HNSCC [57]. Exons v4 and v5 are transcribed together and the present results with separate antibodies for v4 and v5 showing v5 to be present probably indicate that the v4 antibody is unreliable and that the literature contains mostly false negative results.

ICC was performed on cells grown for 2 days, because it was noted that as cells approached confluence, they stained less intensely and for fewer CD44 isoforms, a phenomenon that appears to be specific to keratinocytes [59]. This itself is an interesting finding and suggests that CD44 expression in cell lines tested remains under relatively normal control, possibly linked to differentiation or contact inhibition or that there is progressive synthesis of more heavily glycoslyated forms. The ICC results presented here are in agreement with the published data, but have been obtained with a much wider range of antibodies.

### CD44 expression analysed by western blotting

Blotting, unlike ICC detects both surface CD44 and smaller amounts of CD44 in transit in the cytoplasm. The isoforms detected in positive controls were consistent with the 85–90 kDa N and O linked glycosylated CD44s form, the 180–200 kDa chondroitin sulphate linked CD44s form [60], [61], the 130–160 kDa CD44E form [62] and the 230–250 kDa epican or keratinocyte form known to be present on keratinocytes [63]. All lines stained with most antibodies except the primary-derived line HN22, which failed to stain for v3 and the metastatic cell line OSC-20, which failed to stain for v3 and v6.

There are few published studies with which to compare the present results. Four studies using WB have been performed on OSCC. One group used antibody against the constitutive region of CD44 (Clone E1/E2) and reported that 80% of SCC lines expressed both CD44s and more abundant forms including variant exons [9] consistent with the current work. The second study used antibody only against v3 and claimed to demonstrate a novel v3-containing isoform (85–90 kDa), in addition to higher MW bands, in control and hypopharyngeal HNSCC (FaDu) cell line [64]. In the present work v3 antibody may have stained this new isoform, as shown by a band at 90 kDa, but bands between 120–200 kDa were also observed. The remaining two studies used antibody only against v9 on OSCC lines and detected bands between 70–190 kDa [50], [51]. In the present work, staining with the same clone of v9 antibody produced two major bands at 120–130 kDa, probably indicating the epithelial form and a minor isoform at 180–190 kDa. Bands of MW down to 70 kDa were not seen. However, the 70 kDa band reported cannot represent v9-containing isoform because the MW is too low, further evidence of false positive antibody reactions.

Exon assortment and post translational modification of CD44 variant molecules including glycosylation can mask CD44 exon-specific epitopes and inhibit detection by antibody [58]. Protein lysates used in WB are denatured and may generate false negative results so that negative WB cannot be considered an accurate indication of lack of expression.

### CD44 expression analysed by flow cytometry

FC analysis detects only surface expression of CD44. Most lines stained with many antibodies with the following exceptions; DT and OSC-20 were negative for v4 antibody, HN12, OSC-20, HN30, and HN31 were negative for v10, and most lines were negative for v7 except HN12, HN8, and OSC-19. FC is also susceptible to epitope masking though the target cells are in native configuration. The same antibodies that failed to stain frequently in ICC also failed to stain in FC. There are no published studies on HNSCC cell lines that have used FC to analyse CD44 expression and so the data can only be compared to other antibody techniques.

### Conclusions

From the assessment of multiple transcriptomics data sets in public databases, we have shown that head and neck cancers specifically overexpress *CD44* compared to other solid cancer types, suggesting an important role for *CD44* in these cancers. In addition, compared to tissue specimens, cell line models generally seemed to express lower levels of CD44.

Given the large numbers of *CD44* variant transcripts that can be produced via alternative splicing, we, therefore, investigated primary and metastatic OPSCC cell lines for potential differences in variant transcripts and resulting protein. The range of normal and OPSCC lines tested all transcribed the four common CD44 transcripts v2-10, v3-10, v6-10, and v8-10 except a line termed HN22, which lacked v3-10. Notably, the splicing patterns were the same as those in normal epithelium.

CD44 protein splicing patterns were additionally compared to transcript expression using three independent methods that resulted in increased sensitivity, reduced the number of negative results, and gave broad confirmation of the results. Thus, all CD44 exons identified by PCR were confirmed by at least one of the antibody-based detection methods used. The paired primary and metastasis-derived cell lines showed minimal differences in CD44 splicing, and there were no major differences that correlated with the cell line origin or phenotype.

Overall, this study represents the most comprehensive analysis of CD44 transcription and expression in OPSCC yet performed. Despite *CD44* being highly expressed in comparison to other cancer types, CD44 variant expression does not seem to be fundamentally deranged in OPSCC carcinoma lines. Whilst total CD44 expression is not considered to confer a survival advantage to head and neck cancers, further investigation is warranted to determine whether its variant isoforms might offer a survival advantage or stem cell properties to malignant keratinocytes.
